# Does the use of engorged adult ticks of *Rhipicephalus microplus* as substrate modifies the acaricidal behavior of *Metarhizium anisopliae*?

**DOI:** 10.1007/s10493-025-01003-z

**Published:** 2025-01-30

**Authors:** Agustín Fernández-Salas, Juan Bernardo Romero-Pérez, Miguel Ángel Alonso-Díaz

**Affiliations:** 1 Centro de Enseñanza, Investigación, y Extensión en Ganadería Tropical, Facultad de Medicina Veterinaria y Zootecnia, Universidad, Nacional Autónoma de México, CDMX, México; 2 Escuela de Medicina Veterinaria y Zootecnia, Universidad de Xicotepetl A.C., Puebla, México

**Keywords:** Tick, Fungi, Biological control, Virulence, Entomopathogenic

## Abstract

Entomopathogenic fungi (EPF) is one of the most promising alternatives to regulate tick populations. However, these fungi may lose acaricidal effectiveness over time, due to the storage period and/or successive cultivation on artificial media. It is known that using arthropod pests as a substrate for EPF could potentially alter their acaricidal behavior over time, however, studies using ticks for this purpose are scarce. Hence, the aims of this research were: (1) to isolate strains of *Metarhizium anisopliae* from paddocks of cattle farms, (2) to evaluate the effect of engorged adult ticks of *Rhipicephalus microplus* as a substrate on the tickicide behavior of *Metarhizium anisopliae* strains, and (3) to determine the lethal time of each *M*. *anisopliae* strain to kill 50% (LT50) and 99% (LT99) of engorged ticks. First, the natural acaricidal effect of 10 strains of *M*. *anisopliae sensu lato* isolated from soils of cattle farms on *R. microplus* was evaluated. Second, the influence of substrate (*R*. *microplus*, *Galleria mellonella* and Sabouraud dextrose agar [SDA]) on the acaricidal activity and virulence index (lethal time) of each strain during four generations was evaluated. Strains MaV69, MaV60 and MaV67 showed more than 90% mortality at day 20 post-treatment. The use of engorged adult ticks as substrate increased the virulence of five *M*. *anisopliae s.l.* strains. Larvae of *G*. *mellonella* and SDA as substrate did not modify the acaricidal behavior of the 10 strains evaluated. Seven of ten EPF decreased their LT50 and three decreased their LT99 as an index of their virulence enhancement.

**Supplementary Information:**

The online version contains supplementary material available at 10.1007/s10493-025-01003-z.

## Introduction

The necessity for alternative methods to control *Rhipicephalus microplus* (Canestrini 1888) populations has arisen due to the increasing resistance of this tick to chemical acaricides. Biological control using entomopathogenic fungi (EPF) is one of the most promising alternative options to regulate tick populations (Fernandes et al. 2012). EPF have advantages such as their environmental safety, mass production capacity, and potential impact on the entire tick life cycle (broad spectrum against free-living and parasitic stages) (Alonso-Díaz y Fernández-Salas 2021). This ultimate advantage pertains to their capacity to synthesize proteins that modulate diverse mechanisms of fungal infection (Vidhate et al. 2023) and correspond to their potential to colonize and kill ticks (enzymatic, toxicological and mechanical invasion systems) (Ebani and Mancianti 2021). Despite their many benefits, EPF exhibit variable virulence due to a combination of genetic diversity, environmental factors, and interactions with other microorganisms. For example, Fernández-Salas et al. (2017) showed that out of 55 strains of *Metarhizium anisopliae* isolated from cattle farm paddocks, nine showed high mortality, 20 moderate mortality and 26 low mortalities against *R*. *microplus*. EPF strains, when recently isolated, may not show high mortality but may exhibit other important laboratory characteristics such as rapid growth, high sporulation, appressoria formation or tolerance to high temperature and/or UV rays (Alonso-Díaz et al. 2022, 2024). Moreover, by taking advantage of these biological properties of EPF, there is the possibility of stimulating, improving or reactivating its acaricidal efficacy. One strategy for this purpose is the stimulation of virulence by subculturing using insects or their larvae as substrate (Vandenberg and Cantone 2004). However, reports using ticks for this purpose are scarce, and some studies suggest that it is difficult to transpose results from insects to ticks because of taxonomic/biological difference, high specificity of EPF, and tick tolerance to fungal infection due to physical and structural barriers (Alonso-Díaz y Fernández-Salas 2021; Polar et al. 2005). The objectives of this research were: (1) to isolate strains of *Metarhizium anisopliae* from paddocks of cattle farms, (2) to evaluate the effect of engorged adult ticks of *Rhipicephalus microplus* as a substrate on the tickicide behavior of *Metarhizium anisopliae* strains, and (3) to determine the lethal time of each *M*. *anisopliae s.l.* strains to kill 50% (LT50) and 99% (LT99) of engorged ticks.

## Materials and methods

### Study area and soil sampling

Soils were sampled in 17 cattle production units (CPU) from the center-north zone of the State of Veracruz, Mexico, with heavy livestock activity and high populations of *R*. *microplus* and *Amblyomma mixtum* (Fabricius 1787) ticks. The regional climate is humid tropical with a mean annual temperature of 23.4 ± 0.5 ∘C, an annual rainfall of 1991 ± 392 mm and a relative humidity (RH) of 85% (INEGI 2019).

Soils were obtained from paddocks during the rainy season in the region (May - July). In each cattle farm, a paddock was selected and five soil subsamples of 200–300 g each were collected, homogenized, and deposited in properly identified polyethylene bags (INTA 2000). Subsamples were collected from the ends and center of an ‘‘X’’ with 50 m between subsample points (SENASICA 2012). Samples were taken with a blast-hole soil sampler (Lord 0225^®^, Soilmoisture, CDMX, Mexico) at a depth of 200 mm (INTA 2000) and 30 mm diameter. Samples were transported in plastic coolers for treatment at the Animal Health Laboratory (AHL) of the Teaching, Research and Extension Center for Tropical Livestock situated at Tlapacoyan, Veracruz, México.

### Laboratory soil management and fungal isolation

*Galleria mellonella* bait method was used to isolate EPF from soil samples (Zimmermann 1986). For each soil sample, the sample was moistened with distilled water and passed through a 2 mm sieve to remove rocks, garbage remains, and plant roots; 300 g of sieved soil was deposited in containers; and 5 third instar *G*. *mellonella* larvae were added to bait EPF. Containers were incubated at 27 ± 2 ºC for 8 days and inverted every 2 days to promote contact between larva and soil (Hernández-Velázquez et al. 2011). After incubation, *Galleria* trap larvae were removed from the soil, disinfected with sodium hypochlorite 0.5% for 3 min, washed with distilled water 3 times, and dried with absorbent paper. Larvae were deposited individually in Petri dishes (60 × 10 mm) with Whatman no. 1 filter papers (Neocitec, CDMX, Mexico), and incubated for 10 days at 27 ± 2 ºC and 85–95% relative humidity (RH). Larvae were inspected daily for signs of mycosis, to check the moisture conditions, and to discard pupae and larvae infected with bacteria.

### Identification and collection of entomopathogenic fungi isolates

Fungi grown on *G*. *mellonella* larvae were identified under a microscope with the aid of morphological keys by evaluating their reproductive structures, form and size of the conidia and growth characteristics (Bischoff et al. 2009; Samson et al. 2013). After identification, the fungi characterized as entomopathogenic were sowed on SDA amended 1% yeast extract in test tubes with 500 p.p.m. of chloramphenicol. After 21 days of growth, fungi were re-identified and each EPF strain described was assigned an identification key. Then, the conidia were collected by scraping in distilled water plus 0.1% Tween 80 and stored refrigerated at 4 °C for immediate use in mortality bioassays.

### Obtaining ticks for bioassays

Engorged female ticks of *R*. *microplus* were collected from cattle infested naturally in a CPU in Martinez de la Torre, Veracruz, Mexico. After collection, engorged ticks were transported to the laboratory, disinfected with 1% of sodium hypochlorite, washed 3 times with distilled water, dried with sterile adsorbent paper, and used immediately in the bioassays. After each collection, ticks were taxonomically identified (Bautista-Garfias 2023).

### Assessment of the natural tick mortality caused by EPF

The acaricidal effects of *M*. *anisopliae sensu lato* strains on engorged female ticks and *G*. *mellonella* larvae were evaluated using an adaptation of the immersion test (Drummond et al. 1967). For each *M*. *anisopliae s.l.* strain, 10 engorged female ticks weighing 0.2–0.3 g and ten third instar *G*. *mellonella* larvae were immersed in 10 mL of a suspension of 1 × 10^8^ conidia/mL for 1 min. Ten ticks and ten larvae of *G*. *mellonella* were exposed only to solution (distilled water plus 0.1% Tween 80) without conidia (as a control group). Three replicates were performed for each treatment. After immersion, the ticks and larvae were recovered, placed in Petri dishes and incubated at 27.0 ± 2 ºC and 85–95% RH. *Galleria* larvae were placed with food (Elías-Santos et al. 2008) after exposure. Mortality was recorded every 2 days post-treatment for 20 days if natural mortality in the control group did not exceed 10%. Ticks were considered dead if there was an absence of movement after stimulation, cessation of Malpighian tube movement and by the observation of mycelia emerging from the cuticle. *Galleria mellonella* larvae were considered dead if there was an absence of movement, change of color and by the observation of mycelia emerging from the corpse.

### Initial collection of EPF from ticks and *G*. *mellonella*

20 days post-treatment, spores were recovered from one tick and one larva expressing sporulation from each treated group. Spores were identified according to the isolating organism (*R*. *microplus* or *G*. *mellonella*) and were sowed separately on SDA in quintuplicate for 21 days to obtain a high number of spores.

Concomitantly, another group of spores obtained from *R*. *microplus* were sowed on SDA; this latter group was no longer exposed to ticks or *G*. *mellonella*, but was seeded throughout the experiment in SDA in order to have a control group of spores subjected to exclusive reproduction in artificial medium.

### Second tick mortality assessment

**Fig. 1:**
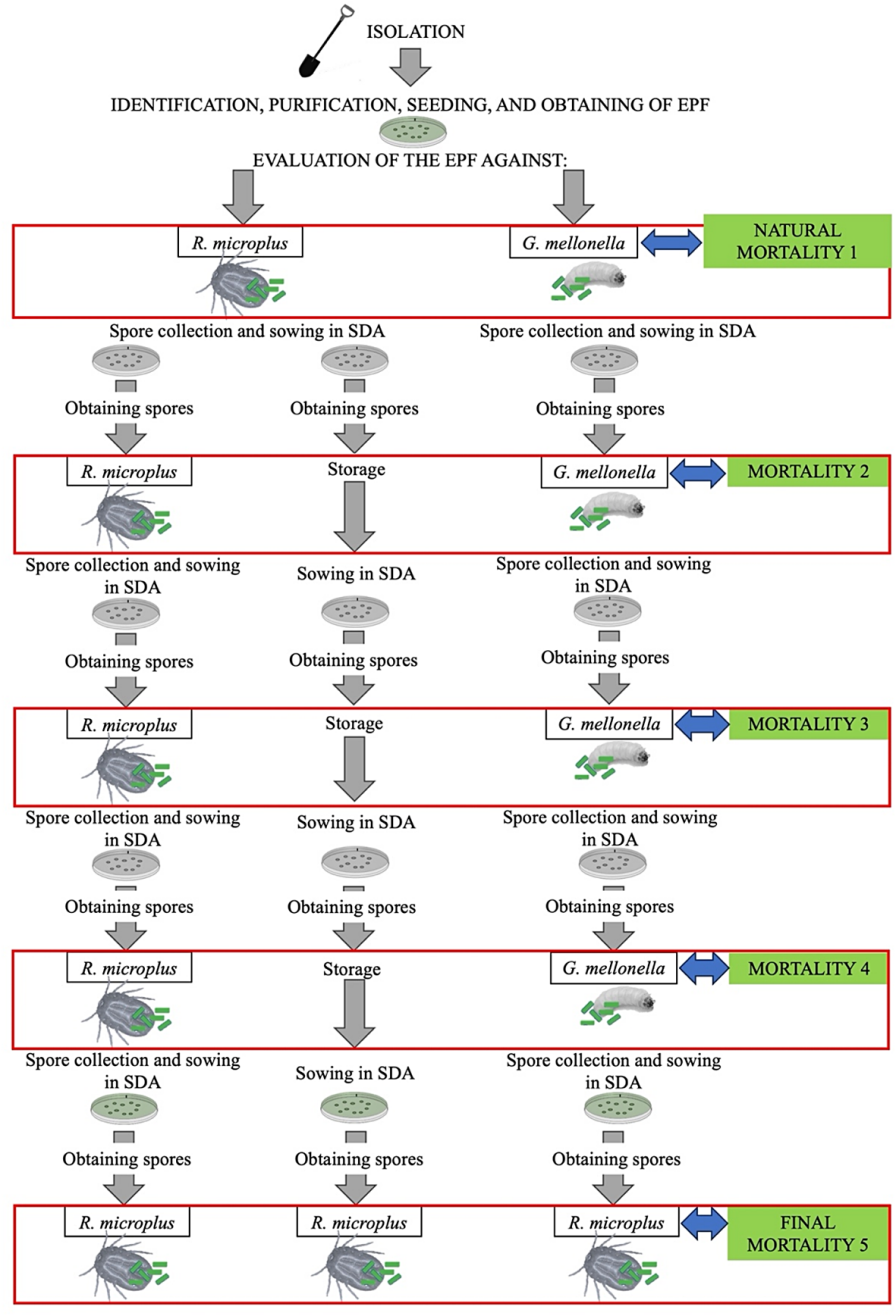
Scheme of serial in vitro transfers of entomopathogenic fungi (EPF) on *Rhipicephalus microplus* ticks, *Galleria mellonella* larvae and on artificial medium (Sabouraud dextrose agar [SDA])

Subsequently, spores sowed from the three groups (ticks, *Galleria* and ADS) were recovered and concentrations were made at 1 × 10^8^ spores/ml. A new group of 10 *R*. *microplus* engorged ticks and 10 *G*. *mellonella* larvae were exposed to the spores obtained. Each group of spores was evaluated against the same organism from which they were isolated. The exposure technique and mortality assessment were performed according to the previously mentioned methodology. This procedure was performed twice more (third and fourth tick mortality assessment), alternating with the collection of EPF (Fig. 1).

### Second collection of EPF from ticks and *G*. *mellonella*

20 days post-treatment, spores were recovered from one tick and one larva expressing sporulation from each treated group. Spores were identified according to the isolating organism (*R*. *microplus* or *G*. *mellonella*) and were sowed separately on SDA in quintuplicate for 21 days to obtain a high number of new spores. This procedure was performed twice more (third and fourth collection of EPF from ticks and *G*. *mellonella*), alternating with the tick mortality assessment (Fig. 1).

### Final (fifth) assessment of *R*. *microplus* mortality

After four serial in vitro transfers, three groups of spores were obtained from each EPF strain: (1) spores obtained from four serial in vitro transfers on *R*. *microplus*, (2) spores obtained from four serial in vitro transfers on *G*. *mellonella*, and (3) spores obtained from four serial in vitro transfers on artificial medium (SDA).

The three spore groups were evaluated against engorged *R*. *microplus* ticks according to the methodology described previously. Mortality evaluation was carried out every two days until day 20. Three replicates were carried out per treatment and a control group consisting of ticks exposed only to a solution of sterile distilled water plus 0.1% Tween 80.

The scheme of the passage methodology to obtain the EPF strains is presented in Fig. 1.

### Statistical analysis

Mortality was calculated using the corrected formula of Abott (1925). The mortality effect of EPF against *R*. *microplus* on natural mortality was assessed using a Kruskal-Wallis test (Statgraphics™ 15.2.06). A one-way analysis of variance was used to assess differences in tick susceptibility to EPF between the five evaluations of the same EPF strain. A Tukey’s HSD test was used as a post hoc analysis to determine significant differences between the means of the five assessments. Calculation of LT50 and LT99 was performed using a probit analysis (Minitab^®^ Sofware). A Chi-Square goodness of fit test was used to determine whether the model was a good fit to the data (normal distribution). A *P* value < 0.05 was considered significant.

## Results

### Identification of entomopathogenic fungi

Ten strains of EPF were isolated and taxonomically identified as *Metarhizium anisopliae sensu lato.*

### Acaricidal effect of EPF against *Rhipicephalus microplus*

The effect of the 10 strains of *M*. *anisopliae s.l.* without any passage on natural mortality of *R*. *microplus* are presented in Table 1. Three strains (MaV60, MaV62 and MaV69) caused ≥ 50% mortality of *R*. *microplus* from day 14 onwards. On day 20 post infection, MaV69, MaV60 and MaV67 induced 100%, 93.3% and 93.3% mortality in *R*. *microplus*, respectively (Table 1).

**Table 1:** Natural mortality caused by 10 strains of *Metarhizium Anisopliae s.l.* without any passage in *Rhipicephalus microplus* engorged females

Strain	Days									
	Mortality (%)									
	**2**	**4**	**6**	**8**	**10**	**12**	**14**	**16**	**18**	**20**
MaV60	0	0	0	3.3	13.3	26.7	50	70	86.7	93.3
MaV61	0	0	10	23.3	33.3	36.7	46.7	50	56.7	63.3
MaV62	0	3.3	16.7	20	33.3	46.7	53.3	66.7	73.3	80
MaV63	0	0	3.3	3.3	6.7	16.7	20	23.3	30	36.7
MaV64	0	0	0	0	13.3	20	26.7	33.3	36.7	36.7
MaV65	0	0	0	13.3	26.7	33.3	36.7	43.3	50	56.7
MaV66	0	0	0	0	0	10	16.7	26.7	30	33.3
MaV67	0	0	0	0	0	16.7	33.3	50	76.7	93.3
MaV68	0	0	0	0	0	6.7	23.3	36.7	50	60
MaV69	0	0	3.3	16.7	33.3	46.7	60	73.3	83.3	100
Control	0	0	0	0	0	3.3	3.3	3.3	6.7	6.7

### Mortality of *Rhipicephalus microplus* caused by EPF after four serial in vitro transfers in engorged ticks

The effect of engorged *R*. *microplus* ticks as substrate on the acaricidal behavior of *Metarhizium anisopliae s.l.* strains is presented in Table 2.

The MaV61, MaV63, MaV65 and MaV69 strains showed no significant changes in mortality on *R*. *microplus* after serial in vitro transfers on these ticks compared to their original natural efficacy (*p* > 0.05, ANOVA, Tukey-HSD) (Table 2). However, strains MaV60, MaV62, MaV64, MaV67 and MaV68 significantly improved their virulence after four serial in vitro transfers using *R*. *microplus* as substrate (*p* < 0.05, ANOVA, Tukey-HSD) (Table 2). MaV66, slightly increased its efficacy in the first 6–10 days (*p* < 0.05), but thereafter mortality was like the original efficacy (*p* > 0.05, ANOVA, Tukey-HSD).

**Table 2:** Average mortality of engorged *R*. *microplus* ticks caused by ten EPF strains of *M*. *Anisopliae s*.*l*. after four serial in vitro transfers on *Rhipicephalus microplus* as substrate *

		Days Mortality (%)													
**Strain**	**Eval.**	**2**	**4**	**6**	**8**		**10**		**12**		**14**		**16**	**18**	**20**
MaV60	1	0^a^	0^a^	0^a^	3.3ª		13.3ª		26.7ª		50ª		70ª	86.7ª	93.3ª
	5	3.3ª	13.3ª	26.7^b^	43.3^b^		53.3^b^		76.7^b^		90^b^		100^b^	100ª	100ª
MaV61	1	0^a^	0^a^	10ª	23.3ª		33.3ª		36.7ª		46.7ª		50ª	56.7ª	63.3ª
	5	0^a^	10^a^	23.3^a^	30ª		30ª		30ª		46.7ª		50ª	66.7ª	73.3ª
MaV62	1	0^a^	3.3ª	16.7ª	20ª		33.3ª		46.7ª		53.3ª		66.7ª	73.3ª	80ª
	5	0^a^	13.3ª	20ª	26.7ª		63.3^b^		83.3^b^		100^b^		100^b^	100^b^	100ª
MaV63	1	0^a^	0^a^	3.3ª	3.3ª		6.7ª		16.7ª		20ª		23.3ª	30ª	36.7ª
	5	0^a^	6.7ª	10ª	20ª		30ª		36.7ª		43.3ª		50ª	53.3ª	66.7ª
MaV64	1	0^a^	0^a^	0^a^	0^a^		13.3ª		20ª		26.7ª		33.3ª	36.7ª	36.7ª
	5	0^a^	16.7^b^	26.7^b^	33.3^b^		36.7ª		53.3^a^		60^b^		66.7^a^	66.7ª	73.3^b^
MaV65	1	0^a^	0^a^	0^a^		13.3ª		26.7ª		33.3ª		36.7ª	43.3ª	50ª	56.7ª
	5	0^a^	6.7^a^	23.3^b^		30ª		36.7^a^		36.7ª		43.3ª	53.3ª	63.3ª	70ª
MaV66	1	0^a^	0^a^	0^a^		0^a^		0^a^		10ª		16.7ª	26.7ª	30ª	33.3ª
	5	0^a^	13.3^a^	23.3^b^		23.3^b^		30^b^		30ª		36.7ª	43.3ª	50ª	56.7ª
MaV67	1	0^a^	0^a^	0^a^		0^a^		0^a^		16.7ª		33.3ª	50ª	76.7ª	93.3ª
	5	3.3^a^	16.7ª	33.3^b^		46.7^b^		60^b^		73.3^b^		100^b^	100^b^	100^b^	100ª
MaV68	1	0^a^	0^a^	0^a^		0^a^		0^a^		6.7ª		23.3ª	36.7ª	50ª	60ª
	5	0^a^	16.7ª	33.3^b^		40^b^		46.7^b^		56.7^b^		73.3^b^	80^b^	86.7^b^	96.7^b^
MaV69	1	0^a^	0^a^	3.3ª		16.7ª		33.3ª		46.7ª		60ª	73.3ª	83.3ª	100ª
	5	0^a^	20ª	26.7^b^		33.3ª		50ª		66.7ª		86.7ª	96.7ª	100ª	100ª

Different literals between lines of each EPF strain indicate significant statistical difference

*Only the first and fifth mortality evaluations are presented in this table. The findings derived from all assessments can be found in the supplementary documentation (refer to Table 2)

### Mortality of Rhipicephalus microplus caused by EPF after four serial in vitro transfers in *Galleria mellonella*

**Fig. 2:**
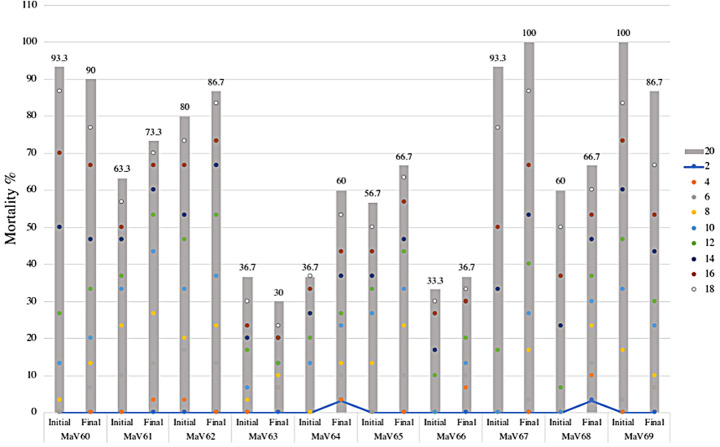
*Rhipicephalus microplus* mortality caused by ten EPF after four serial in vitro transfers on *Galleria mellonella* as substrate. The graph shows the initial mortality obtained in the first evaluation and the final mortality obtained in the fifth evaluation. The colored dots show the temporal mortality recorded every 2 days and the gray bars show the final mortality (day 20) of each evaluation

Figure 2 presents the mortality of engorged *R*. *microplus* ticks caused by EPF after four serial in vitro transfers on *G*. *mellonella* and its comparison with the initial natural mortality.

In summary, the four serial in vitro transfers of *M*. *anisopliae s.l.* strains in *G*. *mellonella* did not influence the acaricidal efficacy of the fungi evaluated against engorged *R*. *microplus* ticks. Only MaV68 strain exhibited a notable acceleration in lethality towards ticks from day 8 to 12 after treatment (*p* < 0.05, ANOVA, Tukey-HSD), however at the end of the observation period (day 20), the mortality rates were similar to the initial natural mortality (*p* > 0.05, ANOVA, Tukey-HSD) (Fig. 2).

### Mortality of *Rhipicephalus microplus* caused by EPF after four serial in vitro transfers on artificial medium

**Fig. 3:**
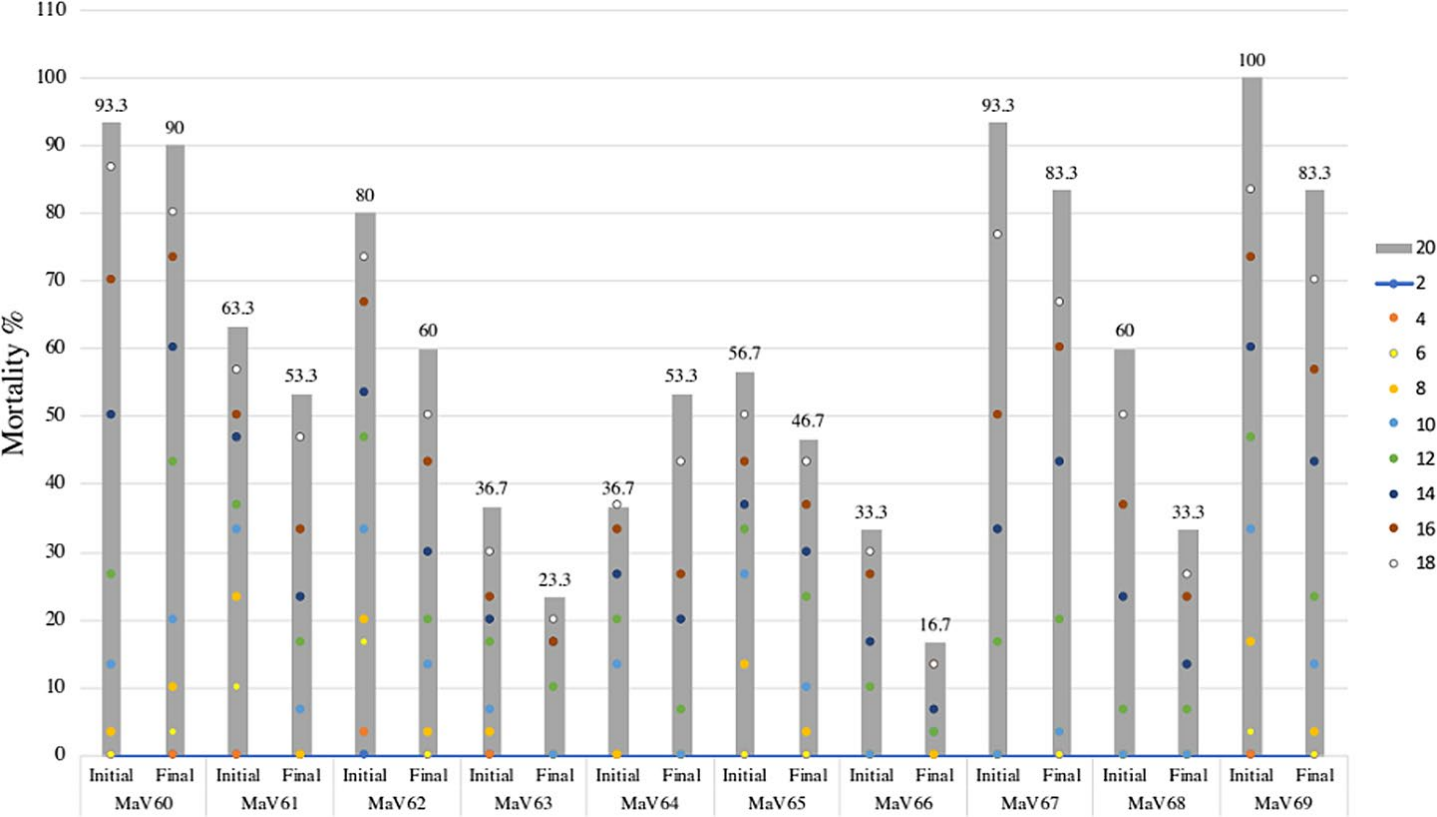
*Rhipicephalus microplus* mortality caused by ten EPF after four serial in vitro transfers on artificial medium as substrate. The graph shows the initial mortality obtained in the first evaluation and the final mortality obtained in the fifth evaluation. The colored dots show the temporal mortality recorded every 2 days and the gray bars show the final mortality (day 20) of each evaluation

Figure 3 presents the mortality of engorged *R*. *microplus* ticks caused by EPF after four serial in vitro transfers on artificial medium and its comparison with the initial natural mortality. The serial in vitro transfers of *M*. *anisopliae s.l.* in artificial medium had no impact on the acaricidal efficacy of the fungi towards *R*. *microplus* (*p* > 0.05, ANOVA, Tukey-HSD). Only MaV62 decreased its tick mortality at days 10 and 12 (*p* < 0.05, ANOVA, Tukey-HSD), however, thereafter, mortalities were similar to the initial natural mortality.

### Lethal times for causing 50% (LT50) and 99% (LT99) mortality of EPF on *R*. *microplus*

Table 3 shows the 50% and 99% lethal times of the ten EPF strains on *R*. *microplus* after four serial in vitro transfers on *R*. *microplus*, *G*. *mellonella* and SDA as substrate. For seven EPF strains (MaV60, MaV62, MaV63, MaV64, MaV67, MaV68 and MaV69), the LT50 decreased significantly after transfers in engorged ticks (*p* < 0.05; Table 3, Probit analysis). In the case of LT99, a similar pattern was observed with three EPF strains (MaV60, MaV62 and MaV67) (*p* < 0.05; Table 3, Probit analysis). The lethal times of EPF serially transferred in *Galleria* and the artificial medium in the fifth evaluation were not significantly modified with respect to the first evaluation (*p* > 0.05; Table 3, Probit analysis).

**Table 3:** Lethal time (days) estimates at 50% and 99% for mortality in *Rhipicephalus microplus* subjected to the adult immersion test with *Metarhizium Anisopliae s.l.* after four serial in vitro transfers on *Rhipicephalus microplus* (rm), *Galleria mellonella* (gm) and artificial medium (AM) as substrate *

Strain	Eval	LT50 (days)	Std. error	CI95%	LT99 (days)	Std. error	CI95%
MaV60	1	14.2ª	0.366	13.5–14.9	22.3ª	0.943	20.8–24.6
	5 (Rm)	8.8^b^	0.383	8.0–9.5	17.8^b^	0.604	16.2–20.0
	5 (Gm)	14.1ª	0.456	13.2–15.0	25.3ª	1.324	23.1–28.5
	5 (AM)	13.5ª	0.418	12.7–14.3	23.7ª	1.136	21.8–26.4
MaV61	1	16.7ª^b^	0.760	14.4–17.5	34.1ª	2.841	29.6–41.5
	5 (Rm)	14.8ª	0.777	13.4–16.6	34.7ª	3.080	31.1–43.0
	5 (Gm)	13.2ª	0.587	12.1–14.5	29.5ª	2.05	26.2–34.7
	5 (AM)	18.6^b^	0.738	17.3–20.4	31.4ª	2.369	27.7–37.8
MaV62	1	13.5^a^	0.555	12.4–14.7	28.5ª	1.839	25.5–33.0
	5 (Rm)	8.8^b^	0.334	8.1–9.4	15.8^b^	0.808	14.5–17.8
	5 (Gm)	12.4ª	0.468	11.5–13.4	24.8ª	1.349	22.6–28.1
	5 (AM)	17.6^c^	0.684	16.4–19.2	31.1ª	2.232	27.6–36.9
MaV63	1	21.9ª	1.571	19.6–26.4	41.1ª^b^	4.798	34.1–55.4
	5 (Rm)	15.9^b^	0.809	14.5–17.8	35.1ª	3.060	30.4–43.2
	5 (Gm)	27.1ª	3.701	22.1–40.6	59.7^b^	11.493	44.4–102
	5 (AM)	28.8ª	1.999	20.9–30.3	41.1^ab^	5.586	33.3–59.9
MaV64	1	20.2ª	1.169	18.4–23.4	37.4ª	3.742	31.8–48.0
	5 (Rm)	12.8^b^	0.701	11.5–14.3	33.1ª	2.840	28.6–40.5
	5 (Gm)	17.3ª	0.943	15.7–19.6	37.0^a^	3.445	31.7–46.3
	5 (AM)	18.9ª	0.659	17.8–20.6	29.4ª	2.10	26.2–35.3
MaV65	1	17.2^ab^	0.814	15.8–19.2	34.3ª	2.857	29.9–41.9
	5 (Rm)	14.8ª	0.790	13.4–16.6	35.3ª	3.195	30.3–43.8
	5 (Gm)	14.9ª	0.666	13.7–16.4	31.9ª	2.411	28.1–38.1
	5 (AM)	18.8^b^	0.902	17.4–21.1	34.4ª	2.957	29.9–42.5
MaV66	1	21.7ª	1.182	19.3–24.5	35.2ª	3.567	30.0–46.0
	5 (Rm)	17.6ª	1.311	15.5–21.1	44.6ª	5.622	36.3–61.1
	5 (Gm)	22.5ª	2.178	19.3–29.2	50.4ª	7.494	39.8–74.3
	5 (AM)	25.8ª	2.715	22.1–36.0	43.4ª	7.078	34.1–70.9
MaV67	1	15.6ª	0.342	15.0–16.4	22.6ª	0.916	21.1–24.9
	5 (Rm)	8.2^b^	0.378	7.5–9.0	16.9^b^	0.942	15.4–19.2
	5 (Gm)	14.1ª	0.403	13.3–15.0	22.8ª	1.081	21.0–25.4
	5 (AM)	15.6ª	0.418	14.8–16.5	24.9ª	1.209	22.9–27.9
MaV68	1	18.1ª	0.541	17.1–19.4	27.8ª^b^	1.733	25.2–32.5
	5 (Rm)	10.4^b^	0.510	9.3–11.4	24.4ª	1.506	22.0–28.1
	5 (Gm)	15.3ª	0.825	13.8–17.2	35.9^b^	3.271	30.8–44.6
	5 (AM)	21.4ª	1.220	19.6–25.0	34.9^b^	3.62	29.7–46.1
MaV69	1	12.7ª	0.402	11.9–13.5	22.6ª^c^	1.064	20.8–25.1
	5 (Rm)	9.3^b^	0.401	8.5–10.1	19.1ª	1.013	17.4–21.5
	5 (Gm)	14.8^c^	0.556	13.8–16.1	28.6^b^	1.783	25.7–33.0
	5 (AM)	15.3^c^	0.441	14.5–16.3	25.4b^c^	1.294	23.3–28.7

Different literals between lines of each EPF strain indicate significant statistical difference

* Only the first and fifth LT50 and LT99 of the mortality evaluations are presented in this table. The findings derived from all assessments can be found in the supplementary documentation (refer to Table 3)

## Discussion

EPF are microorganisms that inhabit the environment, are saprophytes and form a fundamental part of the natural regulation of various organisms, including arthropod pests (Pell 2007). All ten entomopathogenic fungi isolated in the current investigation were classified as *Metarhizium anisopliae sensu lato*, highlighting the high prevalence of this fungus in grassland microenvironments in tropical areas. Some studies have already reported the importance of this fungus in agricultural/livestock systems, mentioning that these soils can be an important natural reservoir of these microorganisms (D’Alessandro et al. 2012; Fernández-Salas et al. 2020). Furthermore, its ability to survive and to persist in adverse environmental conditions has been highlighted (Vänninen 1996), which may explain its frequent presence in intensively managed livestock soils (intensive grazing, trampling, little shade and herbicide application, among others) (Fernández-Salas et al. 2020). The ability of these fungi to colonize livestock soils could be used strategically for integrated pest management, taking advantage of their complex interactions with soil microbiota, vegetation, climate, ticks and cattle, under the premise of sustainable livestock production.

The EPF examined in the current investigation showed a wide array of potential for the control of ticks, illustrating different virulent and pathogenic attributes. This variation has already been described in other studies. For instance, Fernández-Salas et al. (2017) documented mortality rates ranging from 3 to 100% associated with *M*. *anisopliae* fungal applications, while Adames et al. (2011) observed varying levels of efficacy among different strains of this identical fungal species. This disparity can primarily be attributed to the distinct virulence profiles of each strain, its prior interactions with the tick to be controlled, and its capacity to tolerate host immune responses (Fernandes et al. 2011; Kirkland et al. 2004; Perinotto et al. 2012).

In the present study, five strains of *M*. *anisopliae s.l.* significantly increased their virulence after four serial in vitro transfers on engorged *R*. *microplus* ticks. This phenomenon suggests that *M*. *anisopliae* fungi are not only capable of infecting and reproducing on ticks but can also actively adapt to enhance their pathogenicity on ticks. Two previous studies have evidenced the ability of EPF to increase their virulence through successive passes on ticks; for example, Adames et al. (2011) were able to increase mortality by 13.34–17.78% of a strain of *M*. *anisopliae* on *R*. *microplus* after four passes, and Frazzon et al. (2000) reported that the M5 strain of *M*. *anisopliae* increased mortality of *R*. *microplus* from 1.8 to 84% in a single pass. The variance observed in the acaricidal efficacy across various studies may be elucidated by the disparate capacities of entomopathogenic fungi to synthesize a range of toxins and enzymes in varying concentrations (Mora et al. 2017). A pertinent example is the instance of elevated Pr1 enzyme levels, which have been associated with strains that augment their virulence; in contrast, when this virulence is diminished, Pr1 concentrations decline (Ansari and Butt 2011; Shah et al. 2007). Other studies suggest the inheritance to successive generations of altered characteristics involving various genetic mechanisms such as DNA methylation, the activity of transposons and dsRNA viruses (Butt et al. 2006), the production of digestive enzymes, or the ability to evade or suppress tick immune responses. Previous EPF-ticks-environment contact could have stimulated an evolutionary adaptation of fungi to use ticks as substrate, as it has been reported that the acaricidal capacity is also influenced by the place of origin of EPF (Fernández-Salas et al. 2018, 2019; Perinotto et al. 2012) and ticks (Webster et al. 2017). Such investigations help to identify those fungal strains that have acaricidal potential and, when subjected to selection pressure, the EPF with the greatest adaptive capacity are selected. These results possess both theoretical and practical implications, since the use of *M*. *anisopliae* strains previously adapted to ticks could significantly increase the efficacy of biological control under field conditions. However, it is essential to conduct further studies to assess the stability of this enhanced virulence under different environmental scenarios and under different selection pressures.

A key attribute that biological control agents must exhibit is the speed with which they induce mortality in arthropod pests, that constitutes a critical element of fungal virulence (De la Rosa et al. 2002). Although ticks become sick gradually, it is desirable that ticks die within a few days after infection to affect their feeding phase and their ability to oviposit. In the present investigation, it was observed that seven examined strains exhibited a reduction in LT50, while three strains demonstrated a decrease in LT99. To our knowledge, there are no studies in the scientific literature where the lethal times of EPF that were subjected to serial in vitro transfers in ticks have been determined and compared. Most of the research are focused on determining the increase or decrease in tick mortality at a defined time, the growth characteristics of the fungi and their ability to produce enzymes or toxins (Alonso-Díaz and Fernández-Salas 2021). Conversely, in agriculture, the evaluation of EPF lethal times against insect pests has been determined in several studies (Hernández Díaz-Ordaz et al. 2010; Osorio-Fajardo and Canal 2011) and in some it has been reported that serial in vitro transfers on insects increase mortality rates and reduce lethal times (St. Leger et al. 1996; St. Leger and Wang 2010). Hypothetically, factors that could have influenced the decrease in lethal times in the present study were the latency of an intrinsic virulence, mainly because EPF have closely cohabited with ticks in grasslands for a long time. This could be considered as a probable adaptive evolution to ticks through a series of natural selection cycles.

On the other hand, *Galleria mellonella* is one of the most widely used model organisms to evaluate the insecticidal or acaricidal behavior of EPF mainly due to its susceptibility to fungal pathogens (Mukherjee and Vilcinskas 2018). In the current investigation, EPF strains exhibited no noteworthy alterations (whether augmentations or reductions) between initial mortality rates and the mortality rates after four serial in vitro transfers in *G*. *mellonella*. This result is interesting as *G*. *mellonella* could be used as a virulence maintenance substrate with these EPF. It has been reported that *G*. *mellonella* may have the ability to inhibit certain pathogenic characteristics of some EPF and decrease their effect (Dubovskiy et al. 2013; Wrońska et al. 2018), a situation that was not present in this study with these ticks.

Conversely, extensive research has substantiated that persistent EPF cultures on artificial substrates modify both fungal virulence and acaricidal characteristics (Safavi 2012). In the present investigation, the serial in vitro transfers in artificial culture medium (SDA) did not influence the acaricidal behavior of the fungi against *R*. *microplus*. This is an important finding, as it highlights the pathogenic and virulent stability capacity of the fungal strains. The stability in artificial culture media is a desirable feature for large-scale production purposes as its mass production could benefit without affecting its acaricidal quality. Several studies have demonstrated the negative influence of artificial media on the virulence of EPF on some arthropods (Hutwimmer et al. 2008; Lösch et al. 2010; Nahar et al. 2008; Shah et al. 2007), and although other research agrees with this study, it has been reported that this influence depends on each fungal strain and species, and some important ones, such as *M*. *anisoplae* and *Beauveria bassiana*, can remain stable after several serial in vitro transfers (Ansari and Butt 2011; Brownbridge et al. 2001).

## Conclusions

Ten EPF strains identified as *M*. *anisopliae sensu lato* were isolated. Three strains showed high effectiveness for the control of engorged adult *R*. *microplus* ticks. After four serial in vitro transfers of EPF on *R*. *microplus* ticks as substrate, five strains increased their virulence. Fungal serial in vitro transfers on *G*. *mellonella* and Sabouraud dextrose agar did not influence the acaricidal behavior of the fungi against *R*. *microplus*, demonstrating their virulent stability. Seven EPF strains significantly decreased the lethal times 50 and three the LT99 after serial in vitro transfers in ticks.

## Electronic supplementary material

Below is the link to the electronic supplementary material.

Supplementary Material 1

## Data Availability

Data is provided within the manuscript.
