# Supplementary material for: Does the use of engorged adult ticks of Rhipicephalus microplus as substrate modifies the acaricidal behavior of Metarhizium anisopliae?
Source: Exp Appl Acarol. 2025 Jan 30;94(2):34. doi: 10.1007/s10493-025-01003-z (PMC11782390; doi:10.1007/s10493-025-01003-z)
Supplement: Supplementary file 1 — Supplementary Material 1 [file 10493_2025_1003_MOESM1_ESM.docx]

Table 2. Mortality of engorged *R*. *microplus* ticks caused by ten EPF strains of *M*. *anisopliae* during five evaluations (Ev).

|  |  | Days  Mortality [% ± SD (SEM)] | | | | | | | | | |
| --- | --- | --- | --- | --- | --- | --- | --- | --- | --- | --- | --- |
|  |  |  |  |  |  |  |  |  |  |  |  |
| **Strain** | **Ev** | **2** | **4** | **6** | **8** | **10** | **12** | **14** | **16** | **18** | **20** |
| MaV60 | 1 | 0^a^ | 0^a^ | 0^a^ | 3.3ª ± 1.9  (1.10) | 13.3ª ± 3.8  (2.23) | 26.7ª ± 5.1  (2.94) | 50ª ± 6.6  (3.84) | 70ª ± 6.6  (3.84) | 86.7ª ± 5.1  (2.94) | 93.3ª ± 3.8  (2.20) |
|  | 2 | 0^a^ | 0^a^ | 3.3ª^b^ ± 1.9  (1.10) | 10ª ± 0.0  (0.0) | 26.7ª^b^ ± 3.8  (2.20) | 36.7ª ± 3.9  (2.23) | 53.3ª^b^ ± 3.9  (2.23) | 73.3ª ± 6.9  (4.0) | 80ª ± 6.6  (3.84) | 90ª ± 3.3  (1.91) |
|  | 3 | 0^a^ | 6.7ª ± 1.9  (1.10) | 10ª^b^ ± 3.4  (1.93) | 13.3ª^b^ ± 3.9  (2.23) | 20ª^b^ ± 3.3  (1.93) | 43.3ª^b^ ± 5.1  (2.94) | 76.7ª^bc^ ± 3.9  (2.23) | 90ª^b^ ± 3.3  (1.91) | 100ª | 100ª |
|  | 4 | 0^a^ | 13.3ª ± 2  (1.13) | 26.7^b^ ± 1.9 (1.10) | 33.3ª^b^ ± 1.9  (1.10) | 46.7ª^b^ ± 1.9 (1.13) | 63.3ª^b^ ± 2  (2.20) | 83.3^bc^ ± 3.8  (1.10) | 96.7^b^ ± 1.9  (1.10) | 100ª | 100ª |
|  | 5 | 3.3ª ± 1.9 (1.10) | 13.3ª  ± 5.1 (2.94) | 26.7^b^ ± 5.1 (2.94) | 43.3^b^ ± 6.9 (4.0) | 53.3^b^ ± 6.9 (4.0) | 76.7^b^ ± 5.1  (2.94) | 90^c^ ± 0.0 (0.0) | 100^b^ | 100ª | 100ª |
| MaV61 | 1 | 0^a^ | 0^a^ | 10ª^b^ ± 3.4 (1.93) | 23.3ª ± 3.9 (2.23) | 33.3ª ± 3.8 (2.20) | 36.7ª ± 1.9 (1.10) | 46.7ª ± 2.0 (1.13) | 50ª ± 0.0 (0.0) | 56.7ª ± 1.9 (1.10) | 63.3ª ± 1.9 (1.10) |
|  | 2 | 0^a^ | 0^a^ | 0^a^ | 13.3ª ± 2.0 (1.13) | 20ª ± 3.4 (1.93) | 26.7ª ± 3.9 (2.20) | 36.7ª ± 6.9 (4.0) | 43.3ª ± 7.7 (4.43) | 50ª ± 5.8 (3.33) | 53.3ª ± 5.1 (2.94) |
|  | 3 | 0^a^ | 0^a^ | 6.7ª^b^ ± 1.9  (1.10) | 16.7ª ± 2.0  (1.13) | 30ª ± 3.3 (1.91) | 46.7ª ± 3.9 (2.23) | 53.3ª ± 5.1 (2.94) | 56.7ª ± 7.0 (4.02) | 63.3ª ± 5.1 (2.94) | 70ª ± 5.8 (3.33) |
|  | 4 | 0^a^ | 6.7ª ± 3.9 (2.23) | 16.7ª^b^ ± 3.9 (2.23) | 30ª ± 3.3 (1.91) | 46.7ª ± 3.9 (2.23) | 50ª ± 5.8 (3.33) | 56.7ª ± 7.0 (4.02) | 70ª ± 5.8 (3.33) | 73.3ª ± 5.1 (2.94) | 76.7ª ± 6.9 (4.00) |
|  | 5 | 0^a^ | 10^a^ ± 0.0 (0.0) | 23.3^b^ ± 1.9 (1.10) | 30ª ± 3.3 (1.91) | 30ª ± 3.3 (1.91) | 30ª ± 3.3 (1.91) | 46.7ª ± 3.9 (2.23) | 50ª ± 5.8 (3.33) | 66.7ª ± 5.1 (2.94) | 73.3ª ± 5.1 (2.94) |
| MaV62 | 1 | 0^a^ | 3.3ª ± 1.9 (1.10) | 16.7ª ± 2.0 (1.13) | 20ª ± 3.4 (1.93) | 33.3ª ± 1.9 (1.10) | 46.7ª ± 2.0 (1.13) | 53.3ª ± 3.9 (2.23) | 66.7ª ± 5.1 (2.94) | 73.3ª ± 6.9 (4.0) | 80ª ± 5.8 (3.33) |
|  | 2 | 0^a^ | 0^a^ | 10ª ± 3.4 (1.93) | 20ª ± 3.4 (1.93) | 33.3ª ± 1.9 (1.10) | 53.3ª^b^ ± 3.9 (2.23) | 56.7ª ± 5.1 (2.94) | 76.7ª^b^ ± 5.1 (2.94) | 83.3ª^b^ ± 5.1 (2.94) | 96.7ª ± 1.9 (1.10) |
|  | 3 | 0^a^ | 10ª ± 3.4 (1.93) | 23.3ª ± 1.9 (1.10) | 26.7ª ± 1.9 (1.10) | 40ª^b^ ± 3.4 (1.93) | 53.3ª^b^ ± 3.9 (2.23) | 66.7ª^b^ ± 5.1 (2.94) | 80ª^b^ ± 3.4 (1.93) | 93.3ª^b^ ± 1.9 (1.10) | 100ª |
|  | 4 | 0^a^ | 6.7ª ± 1.9 (1.10) | 26.7ª ± 1.9 (1.10) | 40ª ± 3.4 (1.93) | 56.7ª^b^ ± 5.1 (2.94) | 73.3ª^b^ ± 5.1 (2.94) | 86.7ª^b^ ± 5.1 (2.94) | 93.3^b^ ± 3.8 (2.20) | 100^b^ | 100ª |
|  | 5 | 0^a^ | 13.3ª ± 3.9 (2.23) | 20ª ± 3.4 (1.93) | 26.7ª ± 1.9 (1.10) | 63.3^b^ ± 1.9 (1.10) | 83.3^b^ ± 3.9 (2.23) | 100^b^ | 100^b^ | 100^b^ | 100ª |
| MaV63 | 1 | 0^a^ | 0^a^ | 3.3ª ± 1.9 (1.10) | 3.3ª ± 1.9 (1.10) | 6.7ª ± 1.9 (1.10) | 16.7ª ± 2.0 (1.13) | 20ª ± 3.4 (1.93) | 23.3ª ± 5.1 (2.94) | 30ª ± 5.8 (3.33) | 36.7ª ± 6.9 (4.0) |
|  | 2 | 0^a^ | 0^a^ | 0^a^ | 3.3ª ± 1.9 (1.10) | 10ª ± 3.4 (1.93) | 16.7ª ± 2.2 (1.13) | 16.7 ± 2.2 (1.13) | 23.3ª ± 1.9 (1.10) | 33.3ª ± 3.8 (2.20) | 43.3ª ± 2.0 (1.13) |
|  | 3 | 0^a^ | 0^a^ | 6.7ª ± 1.9 (1.10) | 10ª ± 3.4 (1.93) | 16.7ª ± 3.9 (2.23) | 26.7ª ± 5.1 (2.94) | 33.3ª ± 5.1 (2.94) | 43.3ª ± 5.1 (2.94) | 50ª ± 6.7 (3.84) | 56.7ª ± 7.0 (4.02) |
|  | 4 | 3.3^a^ ± 1.9 (1.10) | 3.3ª ± 1.9 (1.10) | 13.3ª ± 3.9 (2.23) | 16.7ª ± 5.1 (2.94) | 23.3ª ± 5.1 (2.94) | 30ª ± 3.3 (1.91) | 36.7ª ± 3.9 (2.23) | 43.3ª ± 5.1 (2.94) | 46.7ª ± 5.1 (2.94) | 53.3ª ± 5.1 (2.94) |
|  | 5 | 0^a^ | 6.7ª ± 1.9 (1.10) | 10ª ± 3.4 (1.93) | 20ª ± 3.4 (1.93) | 30ª ± 5.8 (3.33) | 36.7ª ± 3.9 (2.23) | 43.3ª ± 5.1 (2.94) | 50ª ± 3.4 (1.93) | 53.3ª ± 5.1 (2.94) | 66.7ª ± 3.9 (2.23) |
| MaV64 | 1 | 0^a^ | 0^a^ | 0^a^ | 0^a^ | 13.3ª ± 3.9 (2.23) | 20ª^b^ ± 3.4 (1.93) | 26.7ª ± 3.8 (2.20) | 33.3ª^b^ ± 5.1 (2.94) | 36.7ª ± 5.1 (2.94) | 36.7ª ± 5.1 (2.94) |
|  | 2 | 0^a^ | 0^a^ | 0^a^ | 0^a^ | 6.7ª ± 1.9 (1.10) | 16.7ª ± 3.9 (2.23) | 20ª ± 3.4 (1.93) | 26.7ª ± 1.9 (1.10) | 30ª ± 3.3 (1.91) | 36.7ª ± 3.9 (2.23) |
|  | 3 | 0^a^ | 0^a^ | 6.7ª^b^ ± 3.9 (2.23) | 13.3ª^b^ ± 5.1 (2.94) | 16.7ª ± 6.9 (4.0) | 26.7ª^b^ ± 7.0 (4.02) | 30ª^b^ ± 5.8 (3.33) | 40ª^b^ ± 5.8 (3.33) | 46.7ª ± 6.9 (4.0) | 46.7ª ± 6.9 (4.0) |
|  | 4 | 0^a^ | 0^a^ | 3.3ª ± 1.9 (1.10) | 10ª^b^ ± 3.4 (1.93) | 16.7ª ± 3.9 (2.23) | 26.7ª^b^ ± 3.8 (2.20) | 40ª^b^ ± 3.4 (1.93) | 46.7ª^b^ ± 5.1 (2.94) | 53.3ª ± 5.1 (2.94) | 56.7ª ± 7.0 (4.02) |
|  | 5 | 0^a^ | 16.7^b^ ± 2.0 (1.13) | 26.7^b^ ± 3.8 (2.20) | 33.3^b^ ± 5.1 (2.94) | 36.7ª ± 3.9 (2.23) | 53.3^b^ ± 5.1 (2.94) | 60^b^ ± 3.3 (1.91) | 66.7^b^ ± 5.1 (2.94) | 66.7ª ± 5.1 (2.94) | 73.3^b^ ± 3.9 (2.23) |
| MaV65 | 1 | 0^a^ | 0^a^ | 0^a^ | 13.3ª ± 2.0 (1.13) | 26.7ª^b^ ± 1.9 (1.10) | 33.3ª ± 1.9 (1.10) | 36.7ª ± 3.9 (2.23) | 43.3ª ± 5.1 (2.94) | 50ª ± 5.1 (2.94) | 56.7ª ± 5.1 (2.94) |
|  | 2 | 0^a^ | 6.7^a^ ± 1.9 (1.10) | 10ª^b^ ± 3.4 (1.93) | 16.7ª ± 3.9 (2.23) | 16.7ª ± 3.9 (2.23) | 23.3ª ± 5.1 (2.94) | 30ª ± 5.8 (3.33) | 36.7ª ± 3.9 (2.23) | 46.7ª ± 3.9 (2.23) | 50ª ± 3.4 (1.93) |
|  | 3 | 0^a^ | 3.3^a^ ± 1.9 (1.10) | 3.3ª ± 1.9 (1.10) | 6.7ª ± 1.9 (1.10) | 13.3ª ± 2.0 (1.13) | 20ª ± 3.4 (1.93) | 26.7ª ± 3.8 (2.20) | 36.7ª ± 6.9 (4.0) | 46.7ª ± 6.9 (4.0) | 56.7ª ± 7.0 (4.02) |
|  | 4 | 0^a^ | 3.3^a^ ± 1.9 (1.10) | 10ª^b^ ± 3.4 (1.93) | 16.7ª ± 2.0 (1.13) | 26.7ª^b^ ± 1.9 (1.10) | 33.3ª ± 1.9 (1.10) | 43.3ª ± 2.0 (1.13) | 50ª ± 0.0 (0.0) | 60ª ± 3.3 (1.91) | 73.3ª ± 2.0 (1.13) |
|  | 5 | 0^a^ | 6.7^a^ ± 1.9 (1.10) | 23.3^b^ ± 1.9 (1.10) | 30ª ± 3.3 (1.91) | 36.7^b^ ± 1.9 (1.10) | 36.7ª ± 1.9 (1.10) | 43.3ª ± 2.0 (1.13) | 53.3ª ± 1.9 (1.10) | 63.3ª ± 3.8 (2.20) | 70ª ± 3.4 (1.93) |
| MaV66 | 1 | 0^a^ | 0^a^ | 0^a^ | 0^a^ | 0^a^ | 10ª ± 3.4 (1.93) | 16.7ª ± 2.0 (1.13) | 26.7ª ± 1.9 (1.10) | 30ª ± 3.3 (1.91) | 33.3ª ± 3.8 (2.20) |
|  | 2 | 0^a^ | 0^a^ | 0^a^ | 6.7ª^b^ ± 1.9 (1.10) | 13.3ª^b^ ± 2.0 (1.13) | 13.3ª ± 2.0 (1.13) | 20ª ± 3.4 (1.93) | 33.3ª ± 1.9 (1.10) | 36.7ª ± 1.9 (1.10) | 40ª ± 3.4 (1.93) |
|  | 3 | 0^a^ | 0^a^ | 3.3ª^b^ ± 1.9 (1.10) | 3.3ª^b^ ± 1.9 (1.10) | 10ª^b^ ± 3.4 (1.93) | 23.3ª ± 3.9 (2.23) | 26.7ª ± 1.9 (1.10) | 33.3ª ± 1.9 (1.10) | 33.3ª ± 1.9 (1.10) | 36.7ª ± 1.9 (1.10) |
|  | 4 | 3.3^a^ ± 1.9 (1.10) | 10^a^ ± 3.4 (1.93) | 13.3ª^b^ ± 3.9 (2.23) | 26.7^b^ ± 5.1 (2.94) | 33.3^b^ ± 5.1 (2.94) | 33.3ª ± 5.1 (2.94) | 40ª ± 5.8 (3.33) | 43.3ª ± 7.7 (4.43) | 46.7ª ± 6.9 (4.00) | 46.7ª ± 6.9 (4.00) |
|  | 5 | 0^a^ | 13.3^a^ ± 3.9 (2.23) | 23.3^b^ ± 3.9 (2.23) | 23.3ª^b^ ± 3.9 (2.23) | 30^b^ ± 3.3 (1.91) | 30ª ± 3.3 (1.91) | 36.7ª ± 1.9 (1.10) | 43.3ª ± 5.1 (2.94) | 50ª ± 5.8 (3.33) | 56.7ª ± 7.0 (4.02) |
| MaV67 | 1 | 0^a^ | 0^a^ | 0^a^ | 0^a^ | 0^a^ | 16.7ª ± 3.9 (2.23) | 33.3ª ± 5.1 (2.94) | 50ª ± 3.4 (2.93) | 76.7ª ± 2.0 (1.13) | 93.3ª ± 1.9 (1.10) |
|  | 2 | 0^a^ | 3.3ª ± 1.9 (1.10) | 3.3ª^c^ ± 1.9 (1.10) | 13.3ª^b^ ± 2.0 (1.13) | 36.7^b^ ± 1.9 (1.10) | 43.3^b^ ± 2.0 (1.13) | 66.7^b^ ± 1.9 (1.10) | 80^b^ ± 0.0 (0.00) | 96.7^b^ ± 1.9 (1.10) | 100ª |
|  | 3 | 0^a^ | 0^a^ | 16.7ª^cd^ ± 2.0 (1.13) | 33.3^bc^ ± 1.9 (1.10) | 56.7^b^ ± 5.1 (2.94) | 70^b^ ± 5.8 (3.33) | 76.7^bc^ ± 2.0 (1.13) | 96.7^bc^ ± 1.9 (1.10) | 100^b^ | 100ª |
|  | 4 | 0^a^ | 10ª ± 5.8 (3.33) | 26.7^bc^ ± 5.1 (2.94) | 33.3^bc^ ± 5.1 (2.94) | 53.3^b^ ± 3.9 (2.23) | 66.7^b^ ± 5.1 (2.94) | 76.7^bc^ ± 5.1 (2.94) | 86.7^bc^ ± 3.8 (2.20) | 93.3^b^ ± 1.9 (1.10) | 100ª |
|  | 5 | 3.3^a^ ± 1.9 (1.10) | 16.7ª ± 3.9 (2.23) | 33.3^bd^ ± 3.8 (2.20) | 46.7^c^ ± 2.0 (1.13) | 60^b^ ± 3.3 (1.91) | 73.3^b^ ± 2.0 (1.13) | 100^c^ | 100^c^ | 100^b^ | 100ª |
| MaV68 | 1 | 0^a^ | 0^a^ | 0^a^ | 0^a^ | 0^a^ | 6.7ª ± 1.9 (1.10) | 23.3ª ± 5.1 (2.94) | 36.7ª ± 6.9 (4.0) | 50ª ± 5.8 (3.33) | 60ª ± 5.8 (3.33) |
|  | 2 | 0^a^ | 0^a^ | 0^a^ | 3.3ª ± 1.9 (1.10) | 16.7ª^b^ ± 2.0 (1.13) | 20ª ± 3.4 (1.93) | 33.3ª ± 5.1 (2.94) | 36.7ª ± 3.9 (2.23) | 40ª ± 3.4 (1.93) | 46.7ª ± 3.9 (2.23) |
|  | 3 | 0^a^ | 0^a^ | 10ª^b^ ± 3.4 (1.93) | 13.3ª^b^ ± 3.9 (2.23) | 20ª^b^ ± 3.4 (1.93) | 33.3ª^b^ ± 3.8 (2.20) | 46.7ª^b^ ± 2.0 (1.13) | 50ª^b^ ± 3.4 (1.93) | 56.7ª^b^ ± 3.8 (2.20) | 63.3ª ± 5.1 (2.94) |
|  | 4 | 0^a^ | 6.7ª ± 1.9 (1.10) | 20ª ± 3.4 (1.93) | 36.7^b^ ± 3.9 (2.23) | 43.3^b^ ± 5.1 (2.94) | 60^b^ ± 5.8 (3.33) | 76.7^b^ ± 5.1 (2.94) | 80^b^ ± 6.7 (3.84) | 86.7^b^ ± 5.1 (2.94) | 100^b^ |
|  | 5 | 0^a^ | 16.7ª ± 5.1 (2.94) | 33.3^b^ ± 5.1 (2.94) | 40^b^ ± 6.7 (3.84) | 46.7^b^ ± 6.9 (4.0) | 56.7^b^ ± 5.1 (2.94) | 73.3^b^ ± 5.1 (2.94) | 80^b^ ± 5.8 (3.33) | 86.7^b^ ± 5.1 (2.94) | 96.7^b^ ± 1.9 (1.10) |
| MaV69 | 1 | 0^a^ | 0^a^ | 3.3ª ± 1.9 (1.10) | 16.7ª ± 5.1 (2.94) | 33.3ª^b^ ± 5.1 (2.94) | 46.7ª^b^ ± 5.1 (2.94) | 60ª ± 3.3 (1.91) | 73.3ª^b^ ± 5.1 (2.94) | 83.3ª ± 5.1 (2.94) | 100ª |
|  | 2 | 0^a^ | 0^a^ | 0^a^ | 10ª ± 0.0 (0.0) | 26.7ª ± 1.9 (1.10) | 43.3ª ± 2.0 (1.13) | 56.7ª ± 1.9 (1.10) | 70ª ± 3.4 (1.93) | 93.3ª ± 1.9 (1.10) | 100ª |
|  | 3 | 0^a^ | 6.7ª ± 3.9 (2.23) | 26.7^b^ ± 7.0 (4.02) | 40ª^b^ ± 5.8 (3.33) | 53.3ª^b^ ± 10.7 (6.19) | 63.3ª^b^ ± 8.4 (4.85) | 70ª ± 10 (5.77) | 83.3ª^b^ ± 5.1 (2.94) | 93.3ª ± 3.8 (2.20) | 96.7ª ± 1.9 (1.10) |
|  | 4 | 6.7^a^ ± 3.9 (2.23) | 16.7ª ± 3.9 (2.23) | 33.3^b^ ± 5.1 (2.94) | 56.7^b^ ± 5.1 (2.94) | 66.7^b^ ± 3.9 (2.23) | 80^b^ ± 3.4 (1.93) | 86.7ª ± 3.8 (2.20) | 100^b^ | 100ª | 100ª |
|  | 5 | 0^a^ | 20ª ± 3.4 (1.93) | 26.7^b^ ± 3.8 (2.20) | 33.3ª^b^ ± 5.1 (2.94) | 50ª^b^ ± 6.7 (3.84) | 66.7ª^b^ ± 5.1 (2.94) | 86.7ª ± 1.9 (1.10) | 96.7ª^b^ ± 1.9 (1.10) | 100ª | 100ª |

Different literals between lines of each EPF strain indicate significant statistical difference. SD: standard deviation; SEM: Standard error of the mean.

Table 3. Lethal time (days) estimates at 50% and 99% for mortality in *Rhipicephalus microplus* subjected to the adult immersion test with *Metarhizium anisopliae s.l.*

| Strain | Eval | LT50 (days) | Std. error | CI95% | LT99  (days) | Std. error | CI95% |
| --- | --- | --- | --- | --- | --- | --- | --- |
| MaV60 | 1 | 14.2 | 0.366 | 13.5 – 14.9 | 22.3 | 0.943 | 20.8 – 24.6 |
|  | 2 | 13.7 | 0.426 | 12.8 – 14.5 | 24.1 | 1.794 | 22.2 – 27.9 |
|  | 3 | 11.7 | 0.367 | 10.9 – 12.4 | 20.0 | 0.895 | 18.5 – 22.1 |
|  | 4 | 9.7 | 0.395 | 8.9 – 10.4 | 19.2 | 0.994 | 17.6 – 21.6 |
|  | 5 | 8.8 | 0.383 | 8.0 – 9.5 | 17.8 | 0.604 | 16.2 – 20.0 |
| MaV61 | 1 | 16.7 | 0.760 | 14.4 – 17.5 | 34.1 | 2.841 | 29.6 – 41.5 |
|  | 2 | 17.6 | 0.833 | 16.2 – 19.6 | 34.4 | 2.870 | 29.9 – 42.0 |
|  | 3 | 14.9 | 0.614 | 13.7 – 16.2 | 30.5 | 2.130 | 27.1 – 35.9 |
|  | 4 | 12.8 | 0.588 | 11.7 – 14.0 | 29.4 | 2.061 | 26.1 – 34.5 |
|  | 5 | 14.8 | 0.777 | 13.4 – 16.6 | 34.7 | 3.080 | 31.1 – 43.0 |
| MaV62 | 1 | 13.5 | 0.555 | 12.4 – 14.7 | 28.5 | 1.839 | 25.5 – 33.0 |
|  | 2 | 12.8 | 0.429 | 11.6 – 13.3 | 23.4 | 1.169 | 21.4 – 26.2 |
|  | 3 | 11.1 | 0.443 | 10.2 – 11.9 | 22.7 | 1.211 | 20.7 – 25.6 |
|  | 4 | 9.3 | 0.383 | 8.6 – 10.1 | 18.4 | 0.936 | 16.8 – 20.6 |
|  | 5 | 8.8 | 0.334 | 8.1 – 9.4 | 15.8 | 0.808 | 14.5 – 17.8 |
| MaV63 | 1 | 21.9 | 1.571 | 19.6 – 26.4 | 41.1 | 4.798 | 34.1 – 55.4 |
|  | 2 | 20.8 | 1.242 | 18.9 – 24.2 | 37.5 | 3.839 | 31.8 – 48.6 |
|  | 3 | 17.7 | 0.848 | 16.2 – 19.7 | 34.6 | 2.911 | 30.1 – 42.4 |
|  | 4 | 18.1 | 0.172 | 16.2 – 21.1 | 41.0 | 4.479 | 34.3 – 53.6 |
|  | 5 | 15.9 | 0.809 | 14.5 – 17.8 | 35.1 | 3.060 | 30.4 – 43.2 |
| MaV64 | 1 | 20.2 | 1.169 | 18.4 – 23.4 | 37.4 | 3.742 | 31.8 – 48.0 |
|  | 2 | 21.1 | 1.284 | 19.1 – 24.7 | 37.5 | 3.936 | 31.7 – 49.1 |
|  | 3 | 18.8 | 1.085 | 17.0 – 21.6 | 38.3 | 3.777 | 32.5 – 48.7 |
|  | 4 | 17.2 | 0.752 | 15.8 – 18.9 | 33.0 | 2.563 | 28.9 – 39.7 |
|  | 5 | 12.8 | 0.701 | 11.5 – 14.3 | 33.1 | 2.840 | 28.6 – 40.5 |
| MaV65 | 1 | 17.2 | 0.814 | 15.8 – 19.2 | 34.3 | 2.857 | 29.9 – 41.9 |
|  | 2 | 19.2 | 1.295 | 17.1 – 22.6 | 41.8 | 4.676 | 34.8 – 55.1 |
|  | 3 | 18.5 | 0.926 | 17.0 – 20.8 | 35.4 | 3.072 | 30.6 – 43.6 |
|  | 4 | 15.5 | 0.678 | 14.3 – 17.0 | 31.2 | 2.387 | 28.2 – 38.1 |
|  | 5 | 14.8 | 0.790 | 13.4 – 16.6 | 35.3 | 3.195 | 30.3 – 43.8 |
| MaV66 | 1 | 21.7 | 1.182 | 19.3 – 24.5 | 35.2 | 3.567 | 30.0 – 46.0 |
|  | 2 | 20.5 | 1.235 | 18.6 – 23.8 | 38.2 | 3.916 | 32.3 – 49.4 |
|  | 3 | 20.8 | 1.362 | 18.6 – 24.5 | 40.1 | 4.385 | 33.5 – 52.7 |
|  | 4 | 18.4 | 1.502 | 16.0 – 22.6 | 47.3 | 6.479 | 37.9 – 67.0 |
|  | 5 | 17.6 | 1.311 | 15.5 – 21.1 | 44.6 | 5.622 | 36.3 – 61.1 |
| MaV67 | 1 | 15.6 | 0.342 | 15.0 – 16.4 | 22.6 | 0.916 | 21.1 – 24.9 |
|  | 2 | 12.1 | 0.375 | 11.4 – 12.9 | 20.8 | 0.937 | 19.3 – 23.1 |
|  | 3 | 10.0 | 0.360 | 9.3 – 10.7 | 18.2 | 0.870 | 16.7 – 20.2 |
|  | 4 | 10.1 | 0.433 | 9.2 – 10.9 | 21.1 | 1.122 | 19.3 – 23.8 |
|  | 5 | 8.2 | 0.378 | 7.5 – 9.0 | 16.9 | 0.942 | 15.4 – 19.2 |
| MaV68 | 1 | 18.1 | 0.541 | 17.1 – 19.4 | 27.8 | 1.733 | 25.2 – 32.5 |
|  | 2 | 19.0 | 0.966 | 17.4 – 21.5 | 35.5 | 3.191 | 30.6 – 44.2 |
|  | 3 | 16.2 | 0.714 | 14.9 – 17.8 | 32.7 | 2.506 | 28.7 – 39.1 |
|  | 4 | 10.8 | 0.445 | 9.9 – 11.7 | 22.5 | 1.198 | 20.5 – 25.4 |
|  | 5 | 10.4 | 0.510 | 9.3 – 11.4 | 24.4 | 1.506 | 22.0 – 28.1 |
| MaV69 | 1 | 12.7 | 0.402 | 11.9 – 13.5 | 22.6 | 1.064 | 20.8 – 25.1 |
|  | 2 | 13.0 | 0.363 | 12.4 – 13.8 | 21.3 | 0.919 | 19.8 – 23.5 |
|  | 3 | 10.3 | 0.455 | 9.4 – 11.2 | 22.4 | 1.229 | 20.3 – 25.3 |
|  | 4 | 8.0 | 0.411 | 7.1 – 8.8 | 17.7 | 1.016 | 16.1 – 20.2 |
|  | 5 | 9.3 | 0.401 | 8.5 – 10.1 | 19.1 | 1.013 | 17.4 – 21.5 |

Table 4. *Rhipicephalus microplus* mortality rate caused by EPF after four passages on *Galleria mellonella* as substrate (initial natural mortality vs final mortality).

|  |  | Days  Mortality (%) | | | | | | | | | |
| --- | --- | --- | --- | --- | --- | --- | --- | --- | --- | --- | --- |
|  |  |  |  |  |  |  |  |  |  |  |  |
| **Strain** | **Mortality** | **2** | **4** | **6** | **8** | **10** | **12** | **14** | **16** | **18** | **20** |
| MaV60 | Initial | 0^a^ | 0^a^ | 0^a^ | 3.3ª | 13.3ª | 26.7ª | 50ª | 70ª | 86.7ª | 93.3ª |
|  | Final | 0^a^ | 0^a^ | 6.7^a^ | 13.3^a^ | 20^a^ | 33.3^a^ | 46.7^a^ | 66.7^a^ | 76.7^a^ | 90^a^ |
| MaV61 | Initial | 0^a^ | 0^a^ | 10ª | 23.3ª | 33.3ª | 36.7ª | 46.7ª | 50ª | 56.7ª | 63.3ª |
|  | Final | 0^a^ | 3.3^a^ | 13.3^a^ | 26.7^a^ | 43.3^a^ | 53.3^a^ | 60^a^ | 66.7^a^ | 70^a^ | 73.3^a^ |
| MaV62 | Initial | 0^a^ | 3.3ª | 16.7ª | 20ª | 33.3ª | 46.7ª | 53.3ª | 66.7ª | 73.3ª | 80ª |
|  | Final | 0^a^ | 0^a^ | 13.3^a^ | 23.3^a^ | 36.7^a^ | 53.3^a^ | 66.7^a^ | 73.3^a^ | 83.3^a^ | 86.7^a^ |
| MaV63 | Initial | 0^a^ | 0^a^ | 3.3ª | 3.3ª | 6.7ª | 16.7ª | 20ª | 23.3ª | 30ª | 36.7ª |
|  | Final | 0^a^ | 6.7^a^ | 6.7^a^ | 10^a^ | 13.3^a^ | 13.3^a^ | 20^a^ | 20^a^ | 23.3^a^ | 30^a^ |
| MaV64 | Initial | 0^a^ | 0^a^ | 0^a^ | 0^a^ | 13.3ª | 20ª | 26.7ª | 33.3ª | 36.7ª | 36.7ª |
|  | Final | 3.3ª | 3.3ª | 10ª | 13.3ª | 23.3ª | 26.7ª | 36.7ª | 43.3ª | 53.3ª | 60ª |
| MaV65 | Initial | 0^a^ | 0^a^ | 0^a^ | 13.3ª | 26.7ª | 33.3ª | 36.7ª | 43.3ª | 50ª | 56.7ª |
|  | Final | 0^a^ | 0^a^ | 10^a^ | 23.3ª | 33.3ª | 43.3ª | 46.7ª | 56.7ª | 63.3ª | 66.7ª |
| MaV66 | Initial | 0^a^ | 0^a^ | 0^a^ | 0^a^ | 0^a^ | 10ª | 16.7ª | 26.7ª | 30ª | 33.3ª |
|  | Final | 0^a^ | 6.7ª | 10ª | 13.3ª | 13.3ª | 20ª | 30ª | 30ª | 33.3ª | 36.7ª |
| MaV67 | Initial | 0^a^ | 0^a^ | 0^a^ | 0^a^ | 0^a^ | 16.7ª | 33.3ª | 50ª | 76.7ª | 93.3ª |
|  | Final | 0^a^ | 0^a^ | 3.3ª | 16.7ª | 26.7^b^ | 40ª | 53.3ª | 66.7ª | 86.7ª | 100ª |
| MaV68 | Initial | 0^a^ | 0^a^ | 0^a^ | 0^a^ | 0^a^ | 6.7ª | 23.3ª | 36.7ª | 50ª | 60ª |
|  | Final | 3.3ª | 10ª | 13.3ª | 23.3^b^ | 30^b^ | 36.7^b^ | 46.7ª | 53.3ª | 60ª | 66.7ª |
| MaV69 | Initial | 0^a^ | 0^a^ | 3.3ª | 16.7ª | 33.3ª | 46.7ª | 60ª | 73.3ª | 83.3ª | 100ª |
|  | Final | 0^a^ | 6.7ª | 6.7ª | 10ª | 23.3ª | 30ª | 43.3ª | 53.3ª | 66.7ª | 86.7ª |

Different literals between lines of each EPF strain indicate significant statistical difference.

Table 5. *Rhipicephalus microplus* mortality rate caused by EPF after four passages on artificial medium as substrate (initial natural mortality vs final mortality).

|  |  | Days  Mortality (%) | | | | | | | | | |
| --- | --- | --- | --- | --- | --- | --- | --- | --- | --- | --- | --- |
|  |  |  |  |  |  |  |  |  |  |  |  |
| **Strain** | **Mortality** | **2** | **4** | **6** | **8** | **10** | **12** | **14** | **16** | **18** | **20** |
| MaV60 | Inicial | 0^a^ | 0^a^ | 0^a^ | 3.3ª | 13.3ª | 26.7ª | 50ª | 70ª | 86.7ª | 93.3ª |
|  | Final | 0^a^ | 0^a^ | 3.3ª | 10ª | 20ª | 43.3ª | 60ª | 73.3ª | 80ª | 90ª |
| MaV61 | Inicial | 0^a^ | 0^a^ | 10ª | 23.3ª | 33.3ª | 36.7ª | 46.7ª | 50ª | 56.7ª | 63.3ª |
|  | Final | 0^a^ | 0^a^ | 0^a^ | 0^b^ | 6.7^b^ | 16.7ª | 23.3ª | 33.3ª | 46.7ª | 53.3ª |
| MaV62 | Inicial | 0^a^ | 3.3ª | 16.7ª | 20ª | 33.3ª | 46.7ª | 53.3ª | 66.7ª | 73.3ª | 80ª |
|  | Final | 0^a^ | 0^a^ | 0^a^ | 3.3ª | 13.3^b^ | 20^b^ | 30ª | 43.3ª | 50ª | 60ª |
| MaV63 | Inicial | 0^a^ | 0^a^ | 3.3ª | 3.3ª | 6.7ª | 16.7ª | 20ª | 23.3ª | 30ª | 36.7ª |
|  | Final | 0^a^ | 0^a^ | 0^a^ | 0^a^ | 0^a^ | 10ª | 16.7ª | 16.7ª | 20ª | 23.3ª |
| MaV64 | Inicial | 0^a^ | 0^a^ | 0^a^ | 0^a^ | 13.3ª | 20ª | 26.7ª | 33.3ª | 36.7ª | 36.7ª |
|  | Final | 0^a^ | 0^a^ | 0^a^ | 0^a^ | 0^a^ | 6.7ª | 20ª | 26.7ª | 43.3ª | 53.3ª |
| MaV65 | Inicial | 0^a^ | 0^a^ | 0^a^ | 13.3ª | 26.7ª | 33.3ª | 36.7ª | 43.3ª | 50ª | 56.7ª |
|  | Final | 0^a^ | 0^a^ | 0^a^ | 3.3ª | 10ª | 23.3ª | 30ª | 36.7ª | 43.3ª | 46.7ª |
| MaV66 | Inicial | 0^a^ | 0^a^ | 0^a^ | 0^a^ | 0^a^ | 10ª | 16.7ª | 26.7ª | 30ª | 33.3ª |
|  | Final | 0^a^ | 0^a^ | 0^a^ | 0^a^ | 3.3ª | 3.3ª | 6.7ª | 13.3ª | 13.3ª | 16.7ª |
| MaV67 | Inicial | 0^a^ | 0^a^ | 0^a^ | 0^a^ | 0^a^ | 16.7ª | 33.3ª | 50ª | 76.7ª | 93.3ª |
|  | Final | 0a | 0a | 0a | 3.3ª | 3.3ª | 20ª | 43.3ª | 60ª | 66.7ª | 83.3ª |
| MaV68 | Inicial | 0^a^ | 0^a^ | 0^a^ | 0^a^ | 0^a^ | 6.7ª | 23.3ª | 36.7ª | 50ª | 60ª |
|  | Final | 0a | 0a | 0a | 0a | 0a | 6.7ª | 13.3ª | 23.3ª | 26.7ª | 33.3ª |
| MaV69 | Inicial | 0^a^ | 0^a^ | 3.3ª | 16.7ª | 33.3ª | 46.7ª | 60ª | 73.3ª | 83.3ª | 100ª |
|  | Final | 0a | 0a | 0a | 3.3ª | 13.3ª | 23.3ª | 43.3ª | 56.7ª | 70ª | 83.3ª |

Different literals between lines of each EPF strain indicate significant statistical difference.
